# TLR4 increases the stemness and is highly expressed in relapsed human hepatocellular carcinoma

**DOI:** 10.1002/cam4.2070

**Published:** 2019-04-08

**Authors:** Shuang Zhou, Renle Du, Zhenglu Wang, Wenzhi Shen, Ruifang Gao, Shan Jiang, Yan Fang, Yuzhi Shi, Antao Chang, Lei Liu, Chenghu Liu, Na Li, Rong Xiang

**Affiliations:** ^1^ School of Medicine Nankai University Tianjin China; ^2^ Biobank of Tianjin First Center Hospital Tianjin China; ^3^ Tianjin Key Laboratory of Tumor Microenvironment and Neurovascular Regulation Tianjin China; ^4^ The 2011 Project Collaborative Innovation Center for Biological Therapy Nankai University Tianjin China

**Keywords:** cancer stemness, hepatocellular carcinoma, LPS, SOX2, TLR4

## Abstract

Toll‐like receptor 4 (TLR4) plays an essential role in cancer progress. Here, we find that the expression of TLR4 in relapsed human hepatocellular carcinoma (HCC) clinical samples is higher than that in the non‐relapsed ones, which leads us to explore the role of TLR4 in cancer stemness. We reported that TLR4‐AKT signaling pathway was activated by lipopolysaccharides (LPS) in HCC cell lines to enhance the cancer stemness capacity, which was reflected by the increased percentage of CD133^+^ CD49f^+^ population and side population, enhanced sphere formation, and the upregulation of stemness marker gene‐*SOX2*. Downregulation of *SOX2* attenuated the enhanced HCC stemness induced by LPS, indicating SOX2 as a downstream mediator of LPS‐TLR4 signaling. The role of LPS‐TLR4 signaling in inducing HCC stemness was further confirmed by tumor xenograft experiment in vivo. Taken together, our findings provide a novel therapeutic target to prevent the recurrence of HCC.

## 1 INTRODUCTION

Hepatocellular carcinoma (HCC) is a major problem that challenges human health worldwide.^1^, ^2^ In China, HCC is one of leading causes of cancer‐associated death.^3^ In fact, the treatments for HCC are effective at the early stage. However, most of HCCs are detected at advanced stage when traditional chemotherapy has limited effects, which causes poor prognosis. Relapse of HCC is a big problem after surgical resection and liver transplantation.^4^, ^5^ Because of its poor outcome, mechanistic study of HCC and identification of novel therapeutic target are urgently needed.

Cancer stem cells (CSCs) are characterized by tumor‐initiating, self‐renewal, multiple cell division, and the ability of malignant proliferation.^6^, ^7^ CSCs contribute to the functional and phenotypic heterogeneity in many types of cancer.^8^ CSCs have been shown to be associated with tumorigenesis, epithelial‐mesenchymal transition (EMT), metastasis, and chemoresistance.^9^, ^10^, ^11^, ^12^ Emerging evidences suggest that hepatic CSCs play a vital role in the progression of HCC.^9^, ^11^ Further understanding of the molecular mechanisms that regulate CSCs' function may help us to develop advanced strategy targeting HCC tumorigenesis and relapse.

Here, we identify toll‐like receptor 4 (TLR4) as a regulator of stem properties for HCC. TLR4, a pivotal member of TLR family, can be stimulated by several pathogen‐associated molecular patterns (PAMPs), such as LPS, fusion protein, and envelope protein etc.^13^, ^14^, ^15^ TLR4 was recently proposed as a multi‐functional receptor implicated in inflammatory signaling. Mechanistically, with the help of CD14/MD2, recognition of LPS leads to the dimerization of TLR4, which initiates signaling cascade through either myeloid differentiation primary response gene 88 (MyD88)‐dependent pathway or MyD88‐independent pathway.^16^, ^17^ It was reported that mitogen‐activated protein (MAP) kinase pathways, NF‐κB signaling, and PI3K‐AKT pathway can be activated by both MyD88‐dependent and independent pathway.^15^, ^18^, ^19^, ^20^ Previous studies have reported that TLR4 is expressed in various types of cancer, such as breast cancer,^21^ lung cancer,^22^colorectal cancer,^23^ and liver cancer.^24^ In addition, a correlation between donor *TLR4* polymorphism and the higher risk of HCC recurrence after liver transplantation was reported.^25^ Moreover, TLR4 was considered as a potential stemness maker of HCC.^26^

In this study, we aimed to analyze the role of TLR4 in HCC relapse and stemness regulation. We found that TLR4 was expressed in some HCC cell lines and played a pivotal role in enhancing their stemness through TLR4‐AKT‐SOX2 pathway.

## 2 MATERIALS AND METHODS

### 2.1 Ethics statement

All mouse experimentations were conducted following the standard operating procedures approved by the Ethics Committee of Nankai University. The human specimen's study was approved by the ethics committee of Tianjin First Center Hospital and Nankai University.

### 2.2 Clinical samples

Samples of cirrhosis liver tissues and HCC tissues were obtained from Biobank of Tianjin First Center Hospital from 2005 to 2010. We selected patients with the following criteria: the HCC patients had been followed up for a minimum of 3 years, and conformed to UCSF criteria.^27^ Tissue samples were divided into three groups: Cirrhosis, HCC unrelapsed, and HCC relapsed. The informed consents were obtained from the patients in all cases. All diagnoses were clinically confirmed by imaging and pathological examination.

### 2.3 Cell culture

SK‐HEP‐1 (provided by Dr. Reisfeld, Ralph A, the Scripps Research Institute, CA, USA), HepG2, Hep‐3B, HCCLM3, and SMMC‐7721 cell lines (obtained from the Cell Bank of Chinese Academy of Sciences, Shanghai, China) were cultured in Dulbecco's Modified Eagle Medium (DMEM) supplemented with 1% nonessential amino acid, 10% fetal bovine serum (FBS), 0.1 mg/mL streptomycin, and 100 U/mL penicillin. L02 cells (obtained from the Cell Bank of Chinese Academy of Sciences, Shanghai, China) were maintained in RPMI 1640 supplemented with 10% FBS, 0.1 mg/mL streptomycin, and 100 U/mL penicillin. SMMC‐7721 cells were infected with lentivirus carrying pLV‐H1‐shAKT1‐puro, pLV‐H1‐shSOX2‐puro, or pLV‐H1‐EF1α‐puro expressing scramble shRNA control separately, followed by polyclonal selection by using puromycin to generate stable polyclonal cell line with *AKT1* silencing, *SOX2* silencing, or the shRNA control (SC). SMMC‐7721 cells were infected with lentivirus carrying pLV‐EF1α‐TLR4‐IRES‐Bsd or the pLV‐EF1α‐IRES‐Bsd plasmid (Biosettia, San Diego, CA, USA), followed by polyclonal selection by using blasticidin to generate stable polyclonal cell line with *TLR4* overexpression and the control. ShRNA targeting human SOX2 and the scramble shRNA was described before.^28^ The sequence of shRNA targeting AKT1 (shAKT1) is: 5′‐ AAAAGAATGATGGCACCTTCATTGGTTGGATCCAACCAATGAAGGTGCCATCATTC‐3′. Specific primers used for cloning of human *TLR4* cDNA are: forward primer: 5′‐CGGGATCCATGATGTCTGCCTCGCGC‐3′, reverse primer: 5′‐CGACGCGTTCAGATAGATGTTGCTTCCTGCC‐3′, they were ligated into pLV‐EF1α‐MCS‐IRES‐Bsd expression vector by using the restriction enzymes BamHI and MluI.

### 2.4 Real‐time PCR

Total RNAs were extracted from cells by using TRIzol reagent (Invitrogen, Carlsbad, CA, USA), and then reverse‐transcribed into cDNAs by M‐MLV reverse transcriptase (TransGen Biotech, Beijing, China), according to manufacturer's instructions. The relative expression change of mRNA was determined by real‐time PCR as described before.^28^ The expression of GAPDH was detected as the equal loading control. The primer sequences were listed as follows: 5′‐CCTGTCCCTGAACCCTAT‐3′(forward) and 5′‐AATTCTCCCAGAACCAAA‐3′(reverse) for TLR4; 5′‐GCCTGGGCGCCGAGTGGA‐3′ (forward) and 5′‐GGGCGAGCCGTTCATGTAGGTCTG‐3′(reverse) for SOX2; 5′‐TCTGGACACTGGCTGAATCCT‐3′(forward) and 5′‐CGCTGATTAGGCTCCAACCAT‐3′(reverse) for NANOG; 5′‐GCTCGAGAAGGATGTGGTCC‐3′(forward) and 5′‐CGTTGTGCATAGTCGCTGCT‐3′(reverse) for OCT4; 5′‐TCATTGACCTCAACTACATGGTTT‐3′(forward) and 5′‐GAAGATGGTGATGGGATTTC‐3′ (reverse) for GAPDH.

### 2.5 Western blot

Cell lysates were prepared by using RIPA buffer with addition of protease inhibitor cocktails, phosphatase inhibitor cocktail 2 and 3 (Sigma‐Aldrich, St. Louis, MO, USA) as described previously.^29^ Protein samples were loaded onto 10% Tris‐Acrylamide gels and blotted with primary antibodies including: anti‐TLR4, JNK, SOX2, Caspase 3, and β‐actin (Santa Cruz Biotechnology, Inc., Dallas, TX, USA), anti‐ERK (Epitomics Inc. Burlingame, CA, USA), p‐ERK (ZSGB‐BIO, Beijing, China), anti‐p‐JNK, p38, OCT4, and NANOG (abcam, Cambridge, UK), anti‐MD2 (Bioss Antibodies, Beijing, China), anti‐p‐p38, ATF2, p‐ATF2, AKT, and p‐AKT (Cell Signaling Technology, Danvers, MA, USA) antibodies, and then incubated with proper horseradish peroxidase‐conjugated secondary antibodies (ZSGB‐BIO, Beijing, China). The blots were visualized by using an ECL chemiluminescence kit (Millipore, Billerica, MA, USA).

### 2.6 Fluorescence‐activated cell sorting (FACS) assay

Tumor cells stained with PE‐conjugated mouse anti‐human CD133, APC‐conjugated mouse anti‐human CD49f (BD Biosciences, San Jose, CA, USA), and unstained cells control were subject to flow cytometric analysis. Data were acquired on a flow cytometer (FACSCalibur, BD Biosciences, San Jose, CA, USA) and analyzed with FlowJo software (Tree Star, Inc., Ashland, OR, USA).

### 2.7 Apoptosis assay

Apoptosis was analyzed using the AnnexinV‐fluorescein isothiocyanate (FITC) and propidium iodide (PI) staining kit (BD Biosciences, San Jose, CA, USA) following the manufacturer's instruction. Samples were analyzed by using a FACSCalibur flow cytometer (BD Biosciences, San Jose, CA, USA).

### 2.8 Side population analysis

Cells were suspended at 1 × 10^6^/mL in prewarmed (37°C) phosphate‐buffered saline (PBS) supplemented with 1% FBS. Hoechst 33342 (Sigma‐Aldrich, St. Louis, MO, USA) was added to a final concentration of 5 μg/mL with or without 5 μmol L^−1^ verapamil (Sigma‐Aldrich, St. Louis, MO, USA), an ABC transporter inhibitor to determine the blockade of fluorescent efflux. Cells were incubated in 37°C water bath for 60 minutes and shaken gently every 10 minutes. After the incubation, cells were washed with PBS (4°C), stained with PI (1 μg/mL), and subjected to flow cytometry analyses by using a FACSAria Fusion flow cytometer (BD Biosciences, San Jose, CA, USA).

### 2.9 Cell sphere formation assay

0.5 × 10^3^ cells were placed in each well of a 48‐well ultra‐low attachment plate (CORNING, Corning, NY, USA) in the serum‐free DMEM medium supplemented with 0.02 μg/mL EGF, 0.02 μg/mL bFGF, and 2% B27 (Invitrogen, Carlsbad, CA, USA). After 14 days of culture, the plate was subjected to microscope image. The number and relative area of tumor spheres (diameter >50 μm) were averaged from 4 to 6 images for each well. The relative area of tumor spheres = sphere area/plate area×100%.

### 2.10 Immunofluorescence

0.9 × 10^4^ cells were plated on glass slides in each well of a 24‐well plate for 48 hours. After being washed with precooled PBS, the cells were treated with 4% formaldehyde for 15 minutes. The cells were then blocked with PBS supplemented with 5% goat serum at room temperature for 1 hour and followed by overnight incubation with the primary antibody against SOX2 (abcam) in PBS supplemented with 5% goat serum at 4°C, and then incubated with Alexa Fluor 594 conjugated goat anti‐rabbit IgG secondary antibody (ZSGB‐BIO, Beijing, China) for 2 hours at room temperature. The cells were counterstained with DAPI and visualized by using a confocal microscope (Olympus, Tokyo, Japan).

### 2.11 Immunohistochemistry (IHC)

Paraffin‐embedded sections of human or murine liver cancer tissues were deparaffinized with xylene. Antigen retrieval was performed by using citrate buffer (pH = 6) at a temperature of 97°C for 20 minutes. After exhaustion of endogenous peroxidase with methanol and hydrogen peroxide, the slides were blocked with 0.3% bovine serum albumin (BSA) in 0.1 mol L^−1^ tris‐buffered saline for 30 minutes at room temperature, and incubated with the primary antibody against TLR4, SOX2 (Santa Cruz Biotechnology, Inc., Dallas, Texas, USA), p‐AKT (Cell Signaling Technology, Danvers, MA, USA) overnight at 4°C. Then incubated with proper secondary antibody, and subsequently stained with DAB kit (ZSGB‐BIO, Beijing, China). Tissue sections were stained with hematoxylin, then dehydrated in alcohol, cleared in xylene. The overall staining score was the multiplication of staining percentage (0%‐100%) and staining intensity on a numerical scale (none = 0, weak = 1, moderate = 2, strong = 3). Immunostaining score for each protein was expressed as median (range) averaged from 10 image fields for each patient.

### 2.12 In vivo tumorigenic assay

Six‐week‐old male NOD/SCID mice (Beijing HFK Bioscience Co. Ltd, Beijing, China) were injected subcutaneously on the left flank with 1 × 10^6^ or 5 × 10^5^ SMMC‐7721 cells, which were treated with PBS, LPS, LY294002, or LPS plus LY294002 for 48 hours, respectively. After 7 days, the mice were intratumorally injected with the above reagents every other day for 2 weeks. Tumor volume (cm^3^) = length × width^2^/2.

### 2.13 Statistical analysis

All data were analyzed by the GraphPad Prism5 software (GraphPad Software, San Diego, CA, USA). Two‐tailed *t* test was used for the comparison between two groups. Data were presented as mean + standard error of mean (SEM) unless otherwise specified. Difference was considered statistically significant at *P *<* *0.05.

## 3 RESULTS

### 3.1 Enhanced expressions of TLR4 and SOX2 in relapsed HCCs

**Figure 1:**
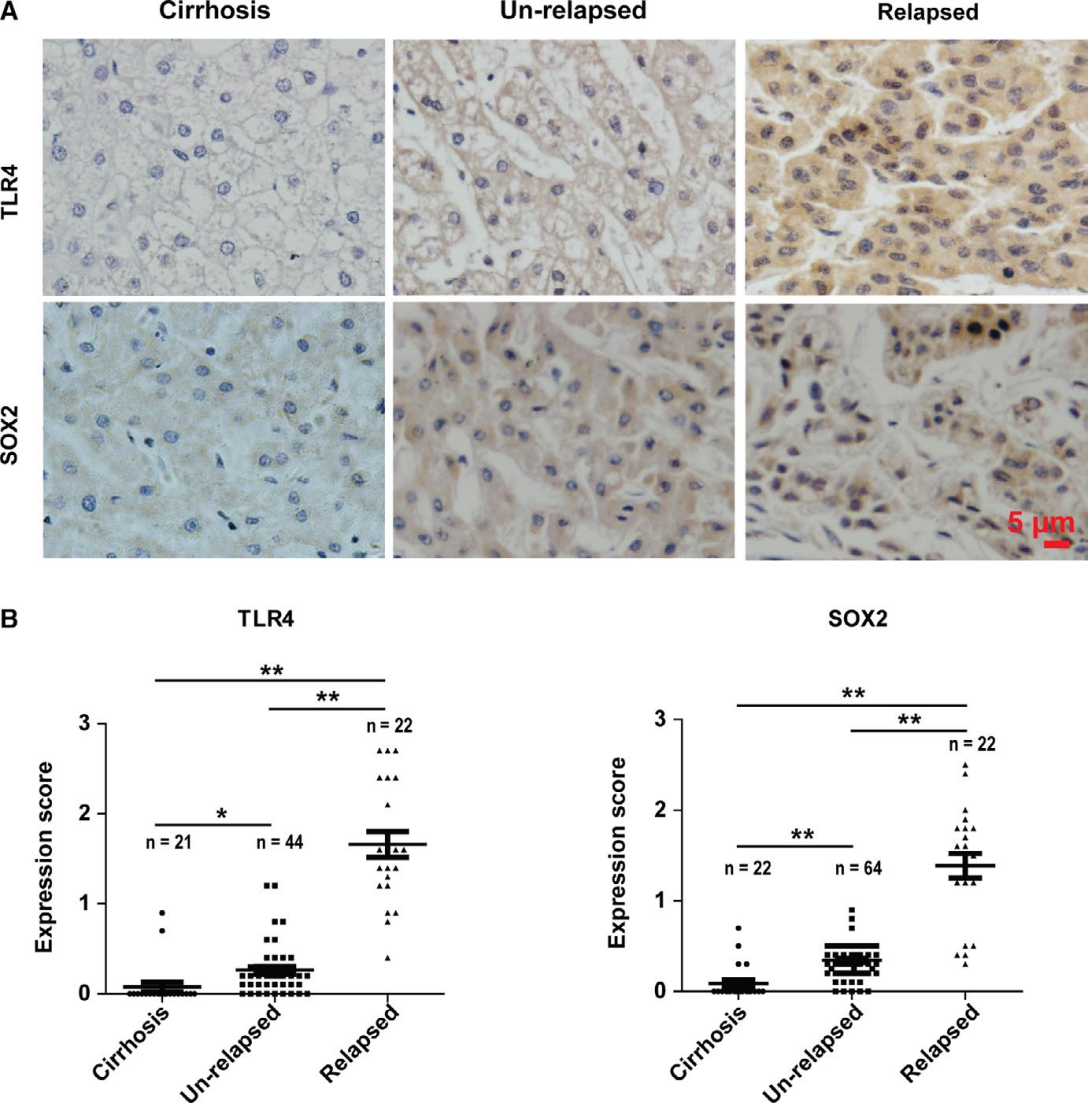
The expressions of TLR4 and SOX2 increase in relapsed HCCs. (A) The representative IHC staining images of TLR4 and SOX2 in cirrhosis and primary HCC lesion samples. (B) The IHC staining scores of TLR4 and SOX2 in different patient groups. Each symbol represents one patient sample. Data are shown as the mean ± SEM. *indicates *P *<* *0.05, **indicates *P *<* *0.01

We detected the expressions of TLR4 and SOX2 by immunohistochemistry in HCC primary lesion samples and cirrhosis samples (Figure 1A). Both protein expression levels of TLR4 and SOX2 were significantly elevated in relapsed HCC samples when compared with the cirrhosis samples and unrelapsed HCC samples (Figure 1B). These findings suggest that enhanced protein expression levels of TLR4 and SOX2 are associated with the relapse of HCC.

### 3.2 Human heptoma cell lines express higher levels of TLR4

**Figure 2:**
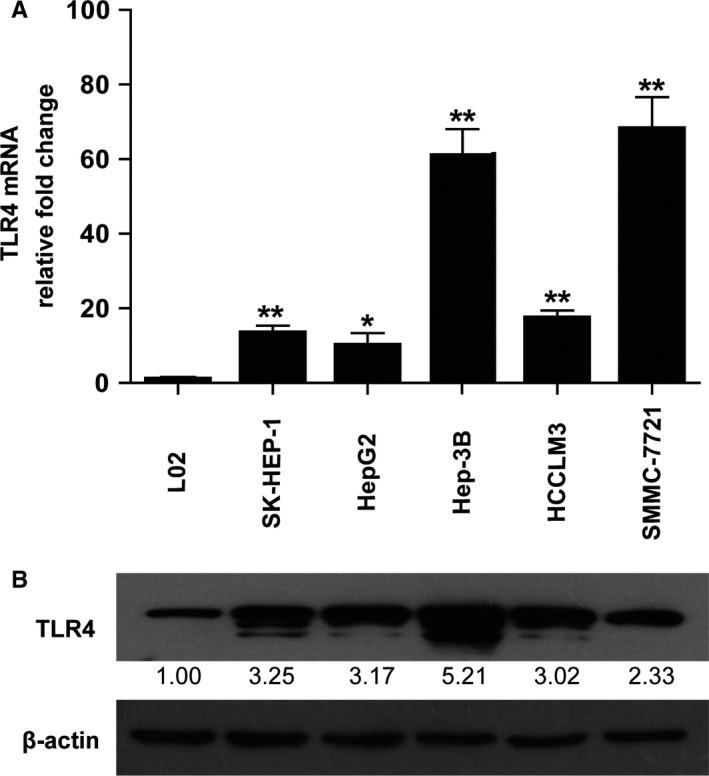
Expression of TLR4 in human liver cell line and hepatoma cell lines. (A) The expression level of *TLR4* was detected at mRNA level by real‐time PCR (A, n = 4‐5) and at protein level by Western blot (B) in human liver cell line (L02), liver adenocarcinoma (SK‐HEP‐1), and hepatocellular carcinoma (HCC) cell lines. The densitometry of each blot was normalized with that of β‐actin and then compared with the normalized control separately to obtain relative fold change (RFC). The mean value of RFC for each blot is indicated at the bottom (n = 3). *indicates *P *<* *0.05, **indicates *P *<* *0.01

We checked the expression of *TLR4* in L02, SK‐HEP‐1, HepG2, Hep‐3B, HCCLM3, and SMMC‐7721 cell lines by real‐time PCR (Figure 2A) and Western blot (Figure 2B). We found that heptoma cell lines expressed relatively higher level of *TLR4* at both mRNA and protein levels when compared with L02, a human liver cell line. Although their mRNA expression levels increased greatly (~10 to 68‐fold), their protein expression levels only increased ~2 to 6‐fold when compared with the L02. The underlying mechanism is complicated, translational and/or posttranslational regulation of *TLR4* in these cell lines may account for this phenomenon.

### 3.3 LPS activates TLR4 signaling pathway in HCC cell lines

**Figure 3:**
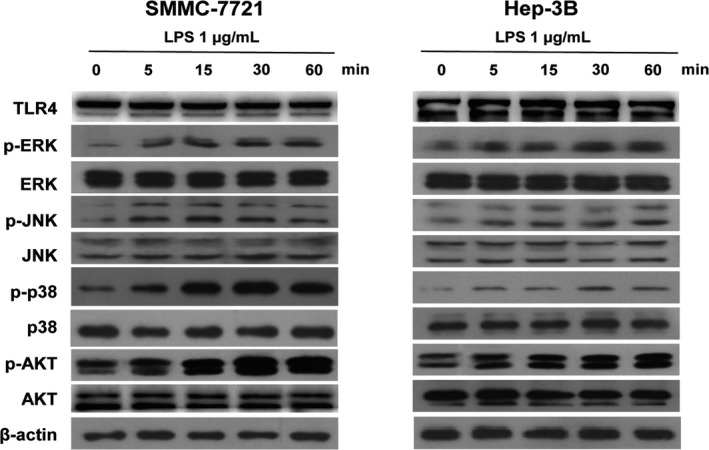
LPS activates TLR4 signaling in human HCC cell lines. Western blot results of TLR4, ERK, JNK, p38, and AKT and the phosphorylation forms of ERK, JNK, p38, and AKT in SMMC‐7721 cells and Hep‐3B cells treated with 1 μg/mL LPS for 0, 5, 15, 30, and 60 minutes (min)

We then examined the function of TLR4 signaling in HCC cell lines. It is widely known that ERK, JNK, p38, and AKT play important roles in LPS‐activated TLR4 signaling pathway. Therefore, we tested the phosphorylation levels of ERK, JNK, p38, and AKT in HCC cell lines treated with LPS (1 μg/mL) by Western blot. In SMMC‐7721 and Hep‐3B cells, the phosphorylation levels of ERK, JNK, p38, and AKT increased after LPS treatment (Figure 3) indicating the activation of TLR4 signaling pathway by LPS in these two cell lines.

### 3.4 LPS activates TLR4 to enhance the stemness of SMMC‐7721 cells and Hep‐3B cells

**Figure 4:**
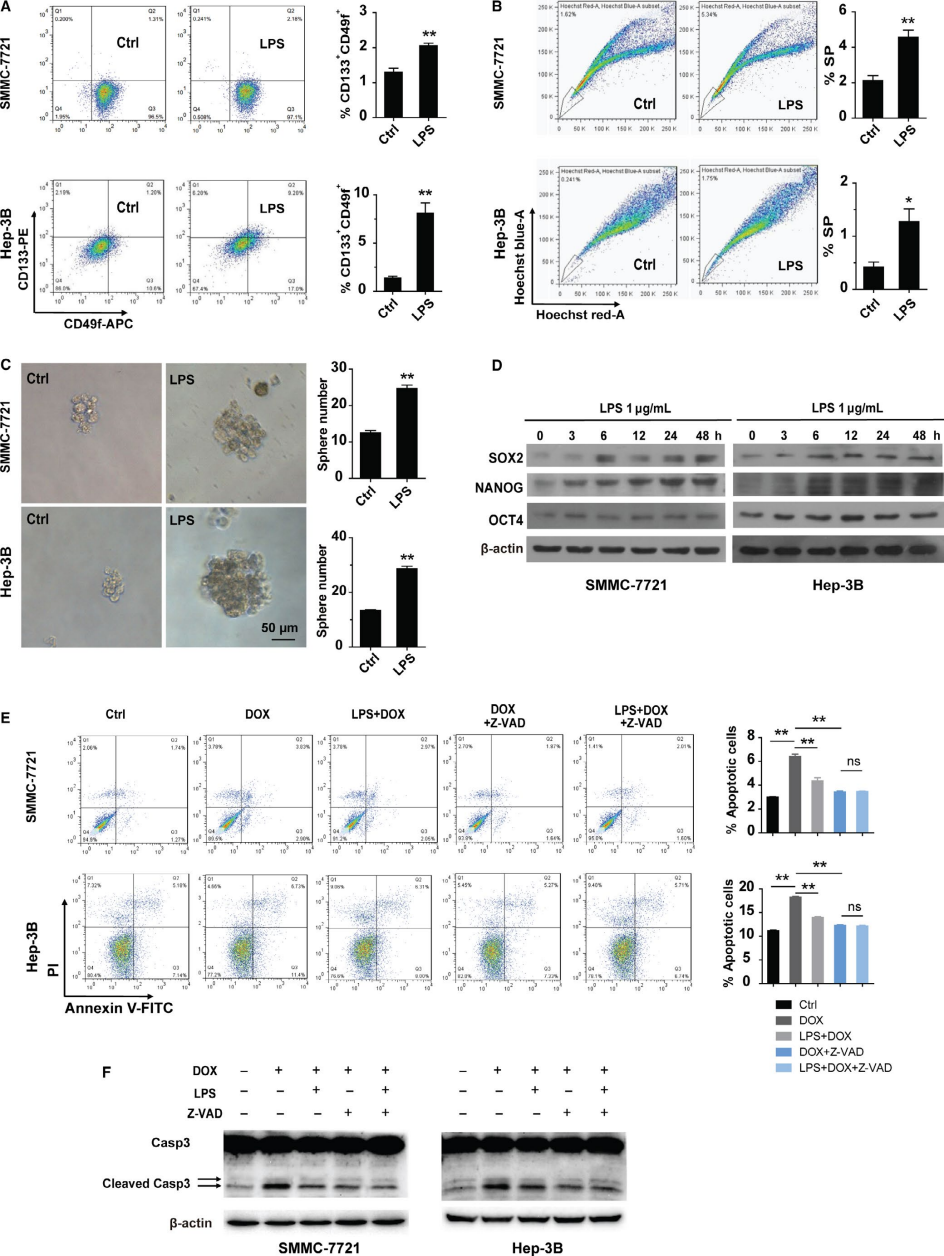
LPS promotes the stemness of HCC cell lines. (A‐B) FACS analyses of CD133^+^ CD49f^+^ cells (A, n = 3 for each group) and the side population (B, n = 3 for each group) in SMMC‐7721 cells and Hep‐3B cells treated with or without LPS for 48 hours. Left panel: representative FACS results, right panel: the statistical results. (C) Sphere formation ability of SMMC‐7721 cells and Hep‐3B cells treated with or without LPS was examined. Left panel: representative images of tumor spheres which were cultured for 14 days, Right panel: the statistical results of sphere number (n = 4 for SMMC‐7721 cells, n = 3 for Hep‐3B cells). (D) Western blot results of SOX2, NANOG, and OCT4 in SMMC‐7721 and Hep‐3B cells treated with LPS for 0, 3, 6, 12, 24, and 48 hours (h). (E) SMMC‐7721 cells and Hep‐3B cells were pre‐incubated with or without LPS for 48 hours and then treated with or without indicated reagents for 24 hours. Left panel: representative FACS results. Right panel: statistical results of apoptotic cells from the FACS (n = 3 for each group). (F) Western blot to show the cleaved caspase 3 (Casp3) in SMMC‐7721 cells and Hep‐3B cells pre‐incubated with or without LPS for 48 hours and then treated with or without indicated reagents for 48 hours. The final concentrations of reagents used in this figure are: LPS: 1 μg/mL, DOX: 0.6 μg/mL, Z‐VAD‐FMK (Z‐VAD): 20 μmol L^−1^. *indicates *P *<* *0.05,**indicates *P *<* *0.01; ns, not significant

Given that SMMC‐7721 cells and Hep‐3B cells can be directly stimulated by LPS, we investigated whether LPS treatment could enhance the stemness of these cells. We treated SMMC‐7721 cells and Hep‐3B cells with or without LPS for 48 hours, and then tested the population changes of CD133^+^CD49f^+^ cells, which represent the CSCs‐like population of HCC.^30^ We found that LPS treatment increased CD133^+^CD49f^+^ cell populations when compared with the untreated controls (Figure 4A). In addition, LPS treatment increased the side population (SP) in these two cell lines (Figure 4B). Sphere formation assay showed that LPS treatment also increased the number of cell spheres in these two cell lines, thus further confirmed the promotion effect of LPS on the stemness of HCC (Figure 4C). To further validate the observation, Western blotting was performed. Increased expression levels of SOX2, NANOG were found in SMMC‐7721 cells and Hep‐3B cells treated with LPS (Figure 4D).

Since chemoresistance is one of the most important characters of CSCs, we examined the chemoresistance activity of SMMC‐7721 and Hep‐3B cells and found that LPS treatment inhibited doxorubicin (DOX)‐induced cell apoptosis and cleavage of caspase 3 (Figure 4E and F). In addition, application of caspase inhibitor‐Z‐VAD‐FMK (Z‐VAD) compromised the apoptosis induced by Dox and diminished the difference of Dox‐induced apoptosis between LPS treatment group and the untreated group, further demonstrating that LPS increased the anti‐apoptosis property of HCCs (Figure 4E and F). Taken together, these data suggest that LPS may activate TLR4 signaling to enhance CSCs‐like populations in these HCC cell lines.

### 3.5 SOX2 is the central downstream mediator of LPS‐TLR4 signaling that results in stemness enhancement

**Figure 5:**
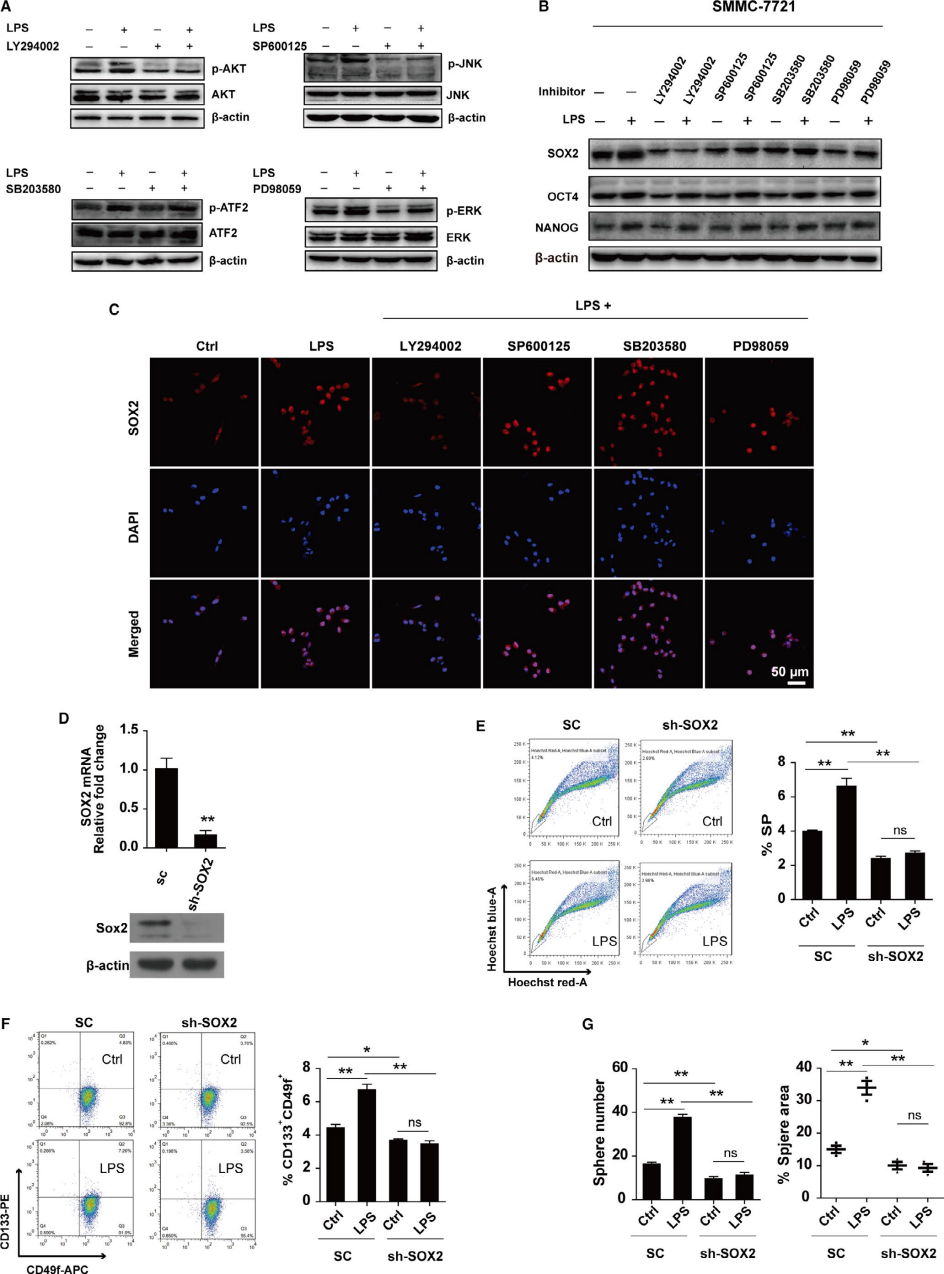
LPS/TLR4 upregulates the expression of SOX2 to enhance the stemness of HCC. (A) Western blot was used to test the phosphorylation changes of AKT, JNK, ATF2, and ERK in SMMC‐7721 cells pre‐incubated with or without indicated inhibitors for 1 hour, and then treated with or without LPS for 30 minutes. (B) Western blot was performed to detect the expression of SOX2, OCT4 and NANOG in SMMC‐7721 cells pre‐incubated with or without indicated inhibitors for 1 hour, and then treated with or without LPS for 48 hours. (C) Immunofluorescence was used to detect the expression of SOX2 in SMMC‐7721 cells pre‐incubated with or without indicated inhibitors for 1 hour, and then treated with or without LPS for 48 hours. (D) The knock‐down efficiency of *SOX2* was examined by real‐time PCR (top panel, n = 3) and Western blot (bottom panel) in SMMC‐7721 cells. (E‐F) The side populations (E, n = 3) and CD133^+^ CD49f^+^ cell populations (F, n = 3) in SMMC‐7721 cells with *SOX2* downregulation (SMMC‐7721‐shSOX2) and control cells (SMMC‐7721‐SC) treated with or without LPS for 48 hours were detected by FACS. Left panel: representative FACS results, right panel: the statistical results from FACS. (G) Statistical results of the sphere number (left panel) and relative sphere area (right panel) in SMMC‐7721 cells treated with or without LPS for 14 days (n = 3 for each group). The final concentrations of reagents used in this figure are: LPS: 1 μg/mL, LY294002: 10 μmol L^−1^, SP600125: 1 μmol L^−1^, SB203580: 10 μmol L^−1^, PD98059: 10 μmol L^−1^. *indicates *P *<* *0.05,**indicates *P *<* *0.01; ns, not significant

LPS‐induced TLR4 signaling has been shown to activate a number of kinases including ERK, JNK, p38, and AKT in various cell types.^15^, ^19^, ^20^, ^31^ To determine which downstream kinase mediates the enhanced effect of LPS on HCC stemness, specific kinase inhibitors were utilized. Before LPS treatment, we pretreated the cells with LY294002, SP600125, SB203580, and PD98059 for 1 hour to inhibit the phosphorylation of AKT, JNK, ATF2 (downstream of p38), and ERK, respectively. We found that the inhibitors effectively reduced the phosphorylation of AKT, JNK, ATF2, and ERK induced by LPS separately (Figure 5A). Remarkably, after treatment with LY294002, a PI3K inhibitor, LPS‐induced expression of the stemness maker gene‐*SOX2* was blocked (Figure 5B).

As a transcription factor, SOX2 plays its transcription‐regulation functions in nucleus. We found when SMMC‐7721 cells were treated with LPS, the expression of nuclear SOX2 increased. When the cells were treated with LPS plus LY294002, the expression level of nuclear SOX2 decreased as compared to LPS treatment alone group. However, when cells were treated with LPS plus other inhibitors, the increased expression of nuclear SOX2 induced by LPS was not obviously reduced as compared to the LPS treatment alone group (Figure 5C). Together, the results show that activated TLR4 may increase the expression of SOX2 via p‐AKT.

Consequently, we knocked down *SOX2* in SMMC‐7721 cells (SMMC‐7721‐shSOX2) (Figure 5D) and found the LPS treatment has no effect on their SP (Figure 5E). Consistently, the population of CD133^+^CD49f^+^ cells in SMMC‐7721‐SC cells increased in response to LPS treatment, but this phenomenon was not observed in SMMC‐7721‐shSOX2 cells (Figure 5F). Similarly, in sphere formation assay, LPS treatment enhanced the sphere number and relative area of SMMC‐7721‐SC cells, but no obvious change was found in SMMC‐7721‐shSOX2 cells when they were treated with LPS (Figure 5G). These data indicate that SOX2 plays a vital role in mediating the stemness‐promotion effect of LPS‐TLR4 signaling in HCC cells.

### 3.6 LPS activates TLR4‐AKT‐SOX2 signaling to promote the stemness of HCC

**Figure 6:**
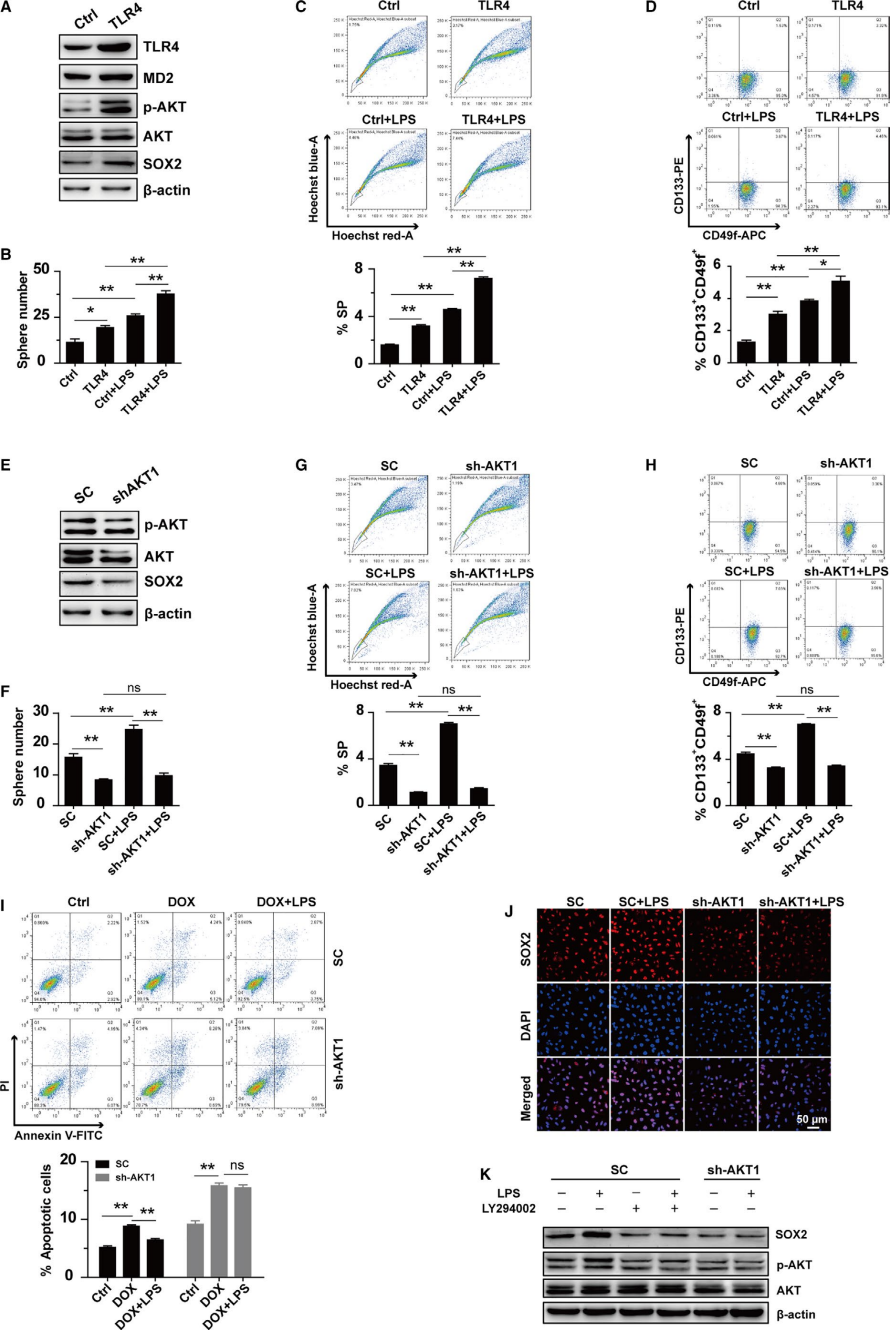
LPS activates TLR4‐AKT‐SOX2 signaling to promote the stemness of HCC. (A) Western blot results of TLR4, MD2, p‐AKT, AKT, and SOX2 in SMMC‐7721 cells. (B‐D) Sphere formation ability (B, n = 3), FACS analysis of side populations (C, n = 3) and CD133^+^ CD49f^+^ cell populations (D, n = 3) in SMMC‐7721 cells with *TLR4* overexpression (SMMC‐7721‐TLR4) and the control cells (SMMC‐7721‐Ctrl) which were treated with or without LPS for 48 hours. (E) Western blot results of p‐AKT, AKT, and SOX2 in SMMC‐7721 cells. (F‐H) Sphere formation ability (F, n = 3), FACS analysis of side populations (G, n = 3) and CD133^+^ CD49f^+^ cell populations (H, n = 3) in SMMC‐7721 cells with *AKT1* silencing (SMMC‐7721‐shAKT1) and the control cells (SMMC‐7721‐SC) treated with or without LPS for 48 hours. (I) Upper panel: representative FACS results in SMMC‐7721 cells that were pre‐incubated with or without LPS for 48 hours and then treated with or without Dox for 24 hours. Lower panel: statistical results for the apoptotic cells from FACS (n = 3). (J) Immunofluorescent staining of SOX2 in SMMC‐7721 cells treated with or without LPS for 48 hours. (K) Western blot results of SOX2, p‐AKT, and AKT in SMMC‐7721 cells pre‐incubated with or without LY294002 for 1 hour and then treated with or without LPS for 48 hours. The final concentrations of reagents used in this figure are: LPS: 1 μg/mL, DOX: 0.6 μg/mL, LY294002: 10 μmol L^−1^. *indicates *P *<* *0.05, **indicates *P *<* *0.01; ns, not significant

To further verify the TLR4‐AKT signaling in mediating the stemness‐promotion effect of LPS, we overexpressed *TLR4* in SMMC‐7721 cells and found that the expressions of p‐AKT and SOX2 were elevated correspondingly (Figure 6A). Sphere formation assay (Figure 6B), Side population assay (Figure 6C) and FACS analysis of CD133^+^CD49f^+^ cell population (Figure 6D) showed that TLR4 increased the stemness of SMMC‐7721 cells. In addition, LPS treatment further increased the stemness property of SMMC‐7721‐TLR4 cells (Figure 6B‐D).

We also knocked down the expression of *AKT1* in SMMC‐7721 cells and found that the expression of SOX2 was downregulated (Figure 6E). Sphere formation assay (Figure 6F), Side population assay (Figure 6G) and FACS analysis of CD133^+^CD49f^+^cell population (Figure 6H) showed that downregulation of *AKT1* decreased the stemness of SMMC‐7721 cells. As expected, silencing of *AKT1* compromised the LPS‐induced chemoresistance and upregulation of SOX2 in SMMC‐7721 cells (Figure 6I‐K). The LPS‐induced upregulation of SOX2 was also compromised when SMMC‐7721‐SC cells were treated with LY294002 (Figure 6K).

### 3.7 LPS enhances stemness of SMMC‐7721 cells in vivo

**Figure 7:**
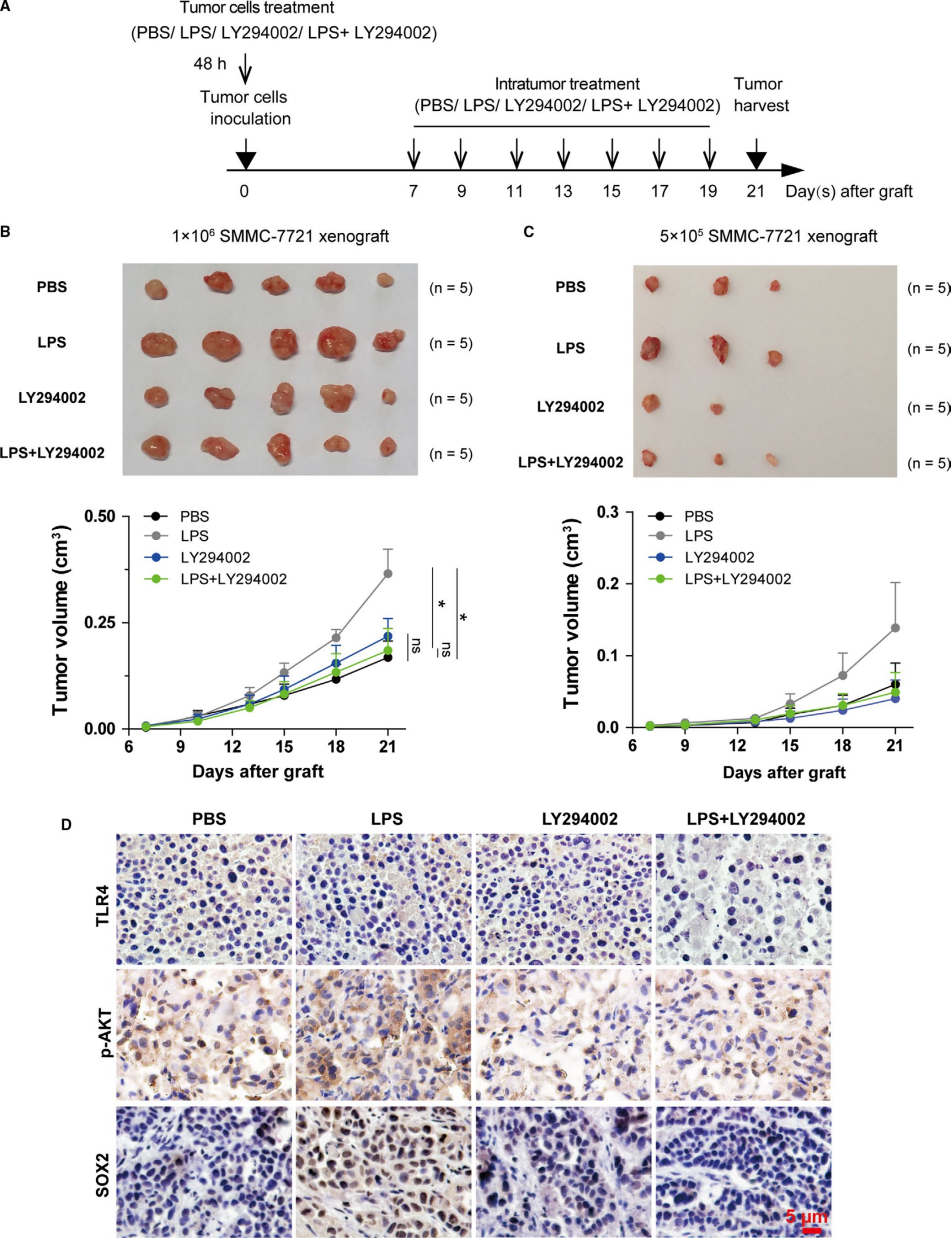
LPS enhances stemness of SMMC‐7721 cells in vivo. (A) Schematic diagram of xenografted HCC mouse model. The final concentrations of reagents used in cell tretament are: LPS: 1 μg/mL, LY294002: 10 μmol L^−1^, (B‐C) Upper panel: tumor xenografts isolated from different recipient NOD/SCID mouse groups intratumorally injected with 100 μL PBS, 100 μL of 5 μg/mL LPS, 100 μL of 50 μmol L^−1^ LY294002, or 50 μL of 10 μg/mL LPS plus 50 μL of 100 μmol L^−1^ LY294002 every other day for two weeks. Lower panel: tumor growth curves of the xenografts for each treatment group. The tumor volumes between different treatment group were compared at the 21st day after graft to test the statistical significance (n = 5 mice for each group). In the 5 × 10^5^ cells inoculation group, the differences between different groups were not compared since many of the values in each group were zero. (D) The representative images showed the immunohistochemistry staining of TLR4, SOX2, and p‐AKT in SMMC‐7721 xenografts in NOD/SCID mice that were treated with PBS, LPS, LY294002, or LPS and LY294002 separately. *indicates *P *<* *0.05, **indicates *P *<* *0.01; ns, not significant

According to the phenomenon that LPS could enhance stemness of HCC cell lines, we analyzed whether LPS could enhance the progression of HCC in vivo. SMMC‐7721 cells were treated with PBS, LPS, LY294002, or LPS plus LY294002 for 48 hours, and then were grafted into NOD/SCID mice. A week after the graft, we injected the above reagents into tumors xenografts every other day for 2 weeks separately (Figure 7A). In the LPS treatment group, the growth of tumors was enhanced when compared with PBS treatment group, but this phenomenon was blocked by LY294002 (Figure 7B), which indicates that LPS promotes stemness through AKT‐mediated downstream pathway in vivo. Limited‐dilution xenograft assay tended to support the stemness‐promotion effect of LPS on SMMC‐7721 cells (Figure 7C). IHC staining showed that LPS did not alter the expression of TLR4, but increased the expressions of p‐AKT and SOX2 (Figure 7D). Together, these observations suggest that LPS induces the activation of TLR4‐AKT to enhance the stemness of HCC in vivo.

## 4 DISCUSSION

As one of the most important pattern recognition receptors (PRRs), TLR4 draws more and more attention for its effect on cancer progression. When activated, TLR4 signal participates in a number of tumor processes, like EMT,^32^ proliferation,^33^ chemoresistance,^34^ and metastasis^35^ etc. Our data show that after LPS treatment, TLR4 was activated to enhance the stemness properties of SMMC‐7721 and Hep‐3B cells, as reflected by increased proportion of CD133^+^CD49f^+^ cells, side population, sphere formation capacity as well as the chemoresistance properties.

Chen.C.L et al reported that TLR4‐NANOG regulated the tumor‐initiating capacity in HCC.^30^ Here, we present evidence that the expression level of SOX2 in HCC cells also increases after treatment with LPS. Therefore, we consider that another pathway besides the TLR4‐NANOG may participate in the stemness regulation in HCC. Recent reports suggest activation of PI3K‐AKT signaling by TLR4.^36^, ^37^ In this study, we found that application of LY294002, which inhibits the phosphorylation of AKT can block the elevated expression of SOX2 induced by LPS. Decreased phosphorylated AKT was reported to promote the expression level of p27,^38^ which directly repressed the transcription of *SOX2*.^39^ In this study, downregulation of *SOX2* by shRNA inhibited the elevation effect of LPS on the proportion of CD133^+^CD49f^+^ cells, side population, and sphere formation capacity, suggesting that besides NANOG, SOX2 is also one of the factors that mediate the stemness‐promotion effect of TLR4 in HCC.

Liver transplantation is one of the most important treatment means for HCC. However, some patients may relapse after receiving the operation, which is a great obstacle for HCC therapy. CSCs are a small population of cells that play a vital role in the development of cancer.^7^ We speculate the stemness of HCC as an important factor responsible for HCC recurrence. As a significant stemness‐regulation protein, the expression of TLR4 is relatively higher in HCC relapse group from our clinic cohort, which conforms to our hypothesis. Portal vein may be a channel through which bacteria or components of bacteria arrive to the liver thus lead to the activation of TLR4 in HCC cells by LPS. Meanwhile, the endogenous ligand of TLR4, high mobility group box 1 (HMGB1) protein, is accumulated in HCC.^40^ These observations make it possible that HCCs with high expression of TLR4 are activated to enhance their stemness property, resulting in the relapse of cancer.

In conclusion, we found that LPS upregulated the expression of SOX2 via TLR4‐AKT pathway, which enhanced the stemness of HCC and thus may increase the risk of relapse after receiving liver transplantation. Finally, we provided a potential therapeutic target, which may improve the outcome of HCC treatment through blocking TLR4‐AKT‐SOX2 signaling.

## CONFLICT OF INTEREST

The authors declare that they have no competing interest.
